# Cyr61 mediates oxaliplatin resistance in colorectal cancer cells by regulating Bcl-xL expression

**DOI:** 10.7150/jca.48891

**Published:** 2021-01-30

**Authors:** Yanfang Song, Yanli Kang, Zhen Lin, Menglu Zeng, Pengchong Shi, Jia Lin, Pingxia Lu, Li Luo, Yingping Cao, Xianjin Zhu

**Affiliations:** 1 Department of Clinical Laboratory, Affiliated People Hospital of Fujian University of Traditional Chinese Medicine, 602 Bayiqi Road, Fuzhou, Fujian 350001, China.; 2 Department of Clinical Laboratory, Fujian Medical University Union Hospital, 29 Xinquan Road, Fuzhou, Fujian 350001, China.; 3 Department of Clinical Laboratory, Fujian Provincial Hospital, 134 Dongjie Road, Fuzhou, Fujian 350001, China.

**Keywords:** cysteine-rich protein 61, colorectal cancer, oxaliplatin, resistance

## Abstract

Although the clinical application of oxaliplatin (L-OHP) has improved the survival of colorectal cancer (CRC) patients, approximately half of patients with CRC fail to achieve good clinical outcomes, indicating resistance to L-OHP therapy. Cysteine-rich protein 61 (Cyr61), a multifunctional extracellular matrix protein, is highly expressed in a variety of tumors; increased Cyr61 expression is known to be closely involved in the chemotherapeutic resistance of many tumors, but its role in the L-OHP resistance of CRC cells has not been studied. In this study, we aimed to investigate the role of Cyr61 in the L-OHP resistance of CRC cells and examine the underlying mechanism. Our findings showed that the mRNA and protein levels of Cyr61 in L-OHP-resistant cells were significantly increased compared with those in nonresistant cells. Knockdown of Cyr61 enhanced the chemosensitivity of L-OHP-resistant cells to L-OHP. Mechanistically, we found that overexpression of Cyr61 decreased L-OHP-induced apoptosis in drug-resistant CRC cells through the regulation of Bcl-xL. Collectively, our results revealed for the first time that Cyr61 plays a crucial role in the resistance of CRC cells to L-OHP and indicated that targeting Cyr61 may be a promising therapeutic strategy to overcome L-OHP resistance in CRC.

## Introduction

Human colorectal cancer (CRC) is one of the most common cancers and is the leading cause of tumor-related death worldwide 1. In recent years, with changes in lifestyle, the morbidity and mortality of CRC have increased in China. Currently, chemotherapy is one of the most common methods of CRC treatment. The third-generation platinum antitumor agent oxaliplatin (L-OHP) is widely used as a first-line chemotherapeutic drug, typically in combination with 5-fluorouracil (5-FU) and leucovorin for CRC treatment. Although clinical application of L-OHP has improved the prognosis of CRC patients, some studies found that approximately half of patients with CRC do not achieve a good clinical therapeutic effect and that their disease continues to progress, indicating that some patients with CRC develop resistance to chemotherapy^2^. Chemoresistance has become a major obstacle in clinical practice; thus, it is necessary to study the mechanism of L-OHP resistance. However, to our knowledge, few studies have focused on resistance to L-OHP, and the mechanism underlying resistance to L-OHP is not yet known. Thus, there is a very urgent need to identify key molecules involved in L-OHP resistance.

Cysteine-rich protein 61 (Cyr61/CCN1) is a member of the CCN (Cyr61/CTGF/NOV) family and can regulate the survival, adhesion, and migration of various normal cells 3. Recently, accumulating evidence has revealed that Cyr61 is highly expressed and that the increased Cyr61 level mediates tumor cell growth and metastasis, as well as drug resistance, in cancers 4-10. In CRC patients, high expression of Cyr61 is found in tumor tissues and serum and is related to tumor growth and metastasis and shorter survival times 11-15. However, the role of Cyr61 in L-OHP resistance in CRC has not yet been reported.

In the present study, we aimed to investigate the contribution of Cyr61 in the resistance of CRC cells to L-OHP and examine the underlying mechanisms. Our findings showed that Cyr61 levels in L-OHP-resistant cells were significantly increased compared with those in nonresistant cells. Knockdown of Cyr61 increased the sensitivity of L-OHP-resistant CRC cells to L-OHP. Mechanistically, we found that overexpression of Cyr61 decreased L-OHP-induced apoptosis in drug-resistant CRC cells through regulation of Bcl-xL. Collectively, these results indicate that Cyr61 plays a crucial role in the resistance of CRC cells to L-OHP and that targeting Cyr61 may be a promising therapeutic strategy to overcome L-OHP resistance in CRC.

## Materials and methods

### Cell culture and establishment of L-OHP-resistant cell lines

Human CRC cell lines (HCT-8 and HCT116) were grown in DMEM medium (Hyclone, Logan, UT, USA) supplemented with 10% fetal bovine serum (Gibco, Carlsbad, CA, USA), 1% (v/v) penicillin/streptomycin (Gibco, Carlsbad, CA, USA) in a humidified CO2 incubator at 37 °C and 5% CO2. To establish resistant cells to L-OHP, HCT-8 and HCT116 cells were cultured in increasing doses of L-OHP (Pharmacia, New Jersey, USA). The established L-OHP-resistant cell lines were named as HCT-8/L-OHP and HCT116/L-OHP, respectively, and were grown in a medium containing 1.6 μg/mL L-OHP unless otherwise indicated. Before subsequent experiments, the cells were cultured in the L-OHP-free medium for at least one week.

### Cell viability assay

To determine the effect of L-OHP on the growth of HCT-8, HCT-8/L-OHP, HCT116 and HCT116/L-OHP cells, these cells were cultured in DMEM medium containing different concentration of L-OHP for 72 h. Cell viability was measured by using Cell Counting Kit-8 (CCK8, Beyotime Biotechnology, Jiangsu, China) according to kit instructions. In brief, 5.0×10^3^ cells were seeded into 96-well plate for 72 h, and 10 μL CCK8 reagents were added into each well for another 2 h. Then, optical density (OD) of plates was determined at 450 nm using a microplate reader (BIO-TEK), and the 50% inhibitory concentration (IC50) values for L-OHP were obtained. Each sample was assayed in triplicate and the experiments were repeated three times.

### Quantitative real-time PCR (qRT-PCR)

To analyze the mRNA level of gene, quantitative real-time PCR was performed as described previously 9-10, 16. In brief, total RNA was extracted from cells using the TRIzol reagent (Invitrogen) according to kit instructions. The cDNA was synthesized from total RNA using the RevertAidTM First Strand cDNA Synthesis Kit (Thermo Scientific, Maryland). Real-time PCR was performed using SYBR Green Master Mix (Applied Biosystems, Foster City, CA). The primers used in this study were described previously 9-10, 16. GAPDH was served as an internal control gene, and the expression of each gene was evaluated using the 2^-∆∆Ct^ method.

### Western blot analysis

To analyze the protein level of Cyr61 and Bcl-xL, western blot analysis was performed as described previously 9-10, 16. In order to block secretion of Cyr61, HCT-8, HCT-8/L-OHP, HCT116, and HCT116/L-OHP cells were treated with Brefeldin A (BD Biosciences, San Jose, CA, 5 μL/ml culture medium) and Monensin (BD Biosciences, San Jose, CA, 2.5 μL/ml culture medium) for 5 h. In brief, total protein was extracted from cells using RIPA lysis buffer (Beyotime Biotechnology, Jiangsu, China). The extracts was separated via 10% SDS-PAGE gel, and then transferred onto PVDF membranes (Millipore, Bedford, MA, USA). Anti-human Cyr61 monoclonal antibody (093G9) (A gift of professor Ningli Li in Shanghai Jiao Tong University School of Medicine, Shanghai, China), anti-GAPDH and anti-Bcl-xL (Cell Signaling Technology, Inc., Beverly, MA) were used in this study. GAPDH was served as an internal control for equal protein loading. For western blotting, each experiment was repeated three times.

### Enzyme-linked immunosorbent assay (ELISA)

To analyze the level of Cyr61 in the supernatant of cell cultures, human Cyr61 enzyme-linked immunosorbent assay (ELISA) kit (R&D Systems, Minneapolis, MN, USA) was used and performed as described previously 9-10, 16.

### Cyr61 knockdown

Cyr61 knockdown was performed as described previously 10. Briefly, lentivirus with shNC or shCyr61 (Shanghai GeneChem Co., Ltd, Shanghai, China) were transfected into HCT-8/L-OHP and HCT116/L-OHP cells. The transduction efficiency of cells was confirmed to be >95% before selection with 4 μg/mL puromycin (Sigma-Aldrich, St. Louis, MO) for 5 days. The knockdown efficiency of Cyr61 was evaluated by western blot analysis.

### Cyr61 overexpression

The lentivirus particles containing the Cyr61 sequence were supported by Shanghai GeneChem Co., Ltd. (Shanghai, China). First, HCT-8 cells (5×10^4^ cells/ml) were cultured in 96-well plates until 70-80% confluency and then transfected with the lentivirus particles containing the Cyr61 sequence for 18-24 h according to the manufacturer's protocol. The transduction efficiency of HCT-8 cells was confirmed to be >95% before selection with 4 μg/mL puromycin (Sigma-Aldrich, St. Louis, MO) for 5 days. The protein level of Cyr61 was determined by western blot analysis.

### Cell apoptosis assay

To detect cell apoptosis, Annexin V-FITC apoptosis detection kit (BD Biosciences, San Jose, CA) was used as described previously 9-10, 16. In brief, the cells were collected and washed with cold PBS buffer, and the supernatant was discarded. Then, the cells (1.0×10^5^) were stained with Annexin V-APC at room temperature for 20 min in the dark. The cell apoptosis rate (Annexin-V positive) was determined by FACSCanto II cytometer (BD Biosciences, San Jose, CA).

### Statistical analysis

SPSS 22.0 statistical software (Version 22.0 SPSS, Chicago, IL; USA) was used for statistical analysis. All experimental data were presented as the mean ± standard error of the mean (SEM) unless indicated. All experiments were repeated at least three times. The significance of differences between groups was assessed by Student's t-test for single comparisons. A value of P < 0.05 was considered to indicate statistical significance.

## Results

### Establishment of HCT-8/L-OHP and HCT116/L-OHP cell lines

**Figure 1:**
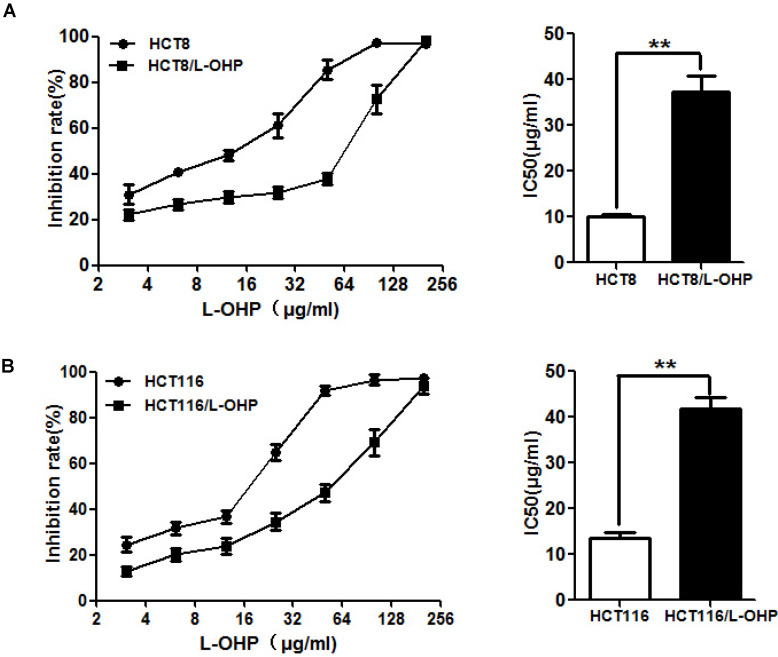
** Evaluation of drug resistance in the HCT-8/L-OHP and HCT116/L-OHP cell lines.** (A) Left panel: HCT-8/L-OHP and parental HCT-8 cells were treated with increasing doses of L-OHP for 72 h and analyzed by a CCK8 assay; Right panel: The IC50 values were calculated using GraphPad Prism 5.0. (B) Left panel: HCT116/L-OHP and parental HCT116 cells were treated with increasing doses of L-OHP for 72 h and analyzed by a CCK8 assay; Right panel: The IC50 values were calculated using GraphPad Prism 5.0. The data are expressed as the mean ± SEM values (n=3). *P < 0.05, **P < 0.01.

To explore the mechanisms of the resistance of CRC cells to L-OHP, we established two L-OHP-resistant CRC cell lines (HCT-8/L-OHP and HCT116/L-OHP). To evaluate the resistance of HCT-8/L-OHP and HCT116/L-OHP cells to L-OHP, a CCK8 assay was used. The results showed that the IC50 value of L-OHP in HCT-8/L-OHP was 37.37±3.332 μg/ml and that the IC50 value of L-OHP in HCT-8 cells was 10.16±0.50 μg/ml, indicating that HCT-8/L-OHP cells exhibited a 3.7-fold increase in resistance to L-OHP compared with HCT-8 cells (Figure 1A); in addition, the IC50 values of L-OHP were 41.81±2.511 μg/ml and 13.43±1.467 μg/ml in HCT116/L-OHP and parental HCT116 cells, respectively, indicating that HCT116/L-OHP cells exhibited a 3.1-fold increase in resistance to L-OHP compared with HCT116 cells (Figure 1B). These results indicated that HCT-8/L-OHP and HCT116/L-OHP cells were more resistant to L-OHP than the corresponding parental HCT-8 and HCT116 cells.

### The level of Cyr61 is significantly increased in L-OHP-resistant CRC cells

**Figure 2:**
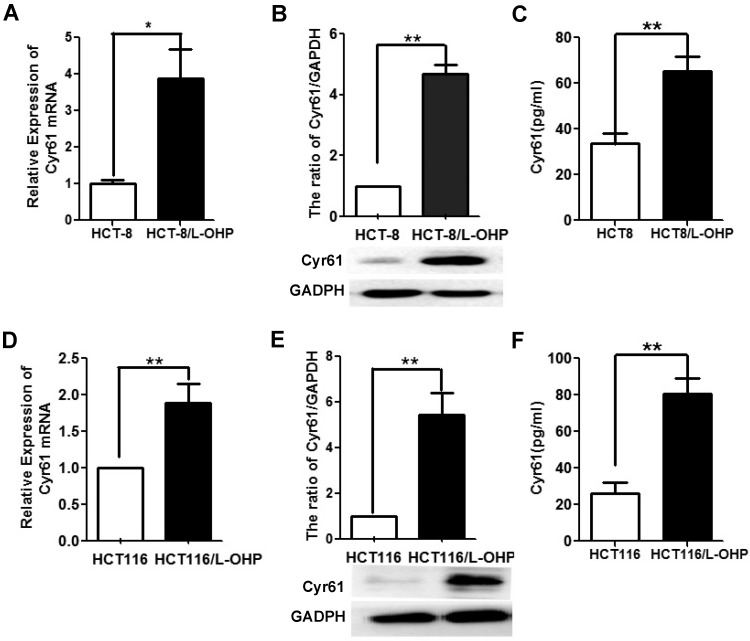
** The level of Cyr61 in the HCT-8/L-OHP and HCT116/L-OHP cell lines.** (A) and (D) The mRNA level of Cyr61 was determined by RT-PCR. (B) and (E) The protein level of Cyr61 was determined by western blotting. (C) and (F) The concentration of Cyr61 in the cell supernatant was measured by ELISA. The data are expressed as the mean ± SEM values (n=3). *P < 0.05, **P < 0.01.

Previous studies have shown that Cyr61 levels in the serum and cancer tissue of CRC patients are upregulated and that high Cyr61 levels suggest a poor prognosis in patients with CRC 11-15. To analyze the relationship between Cyr61 levels and drug resistance in CRC cells, we examined Cyr61 levels in two L-OHP-resistant CRC cell lines. As shown in Figure 2, the mRNA and protein levels of Cyr61 in L-OHP-resistant cells (HCT-8/L-OHP and HCT116/L-OHP) were significantly increased compared with those in the corresponding parental cell lines (HCT-8 and HCT116). Consistent with these results, the Cyr61 levels in the supernatants of the two L-OHP-resistant cell lines were higher than those in the corresponding parental cell lines. Collectively, these results indicate that the level of Cyr61 is significantly increased in L-OHP-resistant CRC cells.

### Cyr61 is involved in the resistance of CRC cells to L-OHP

**Figure 3:**
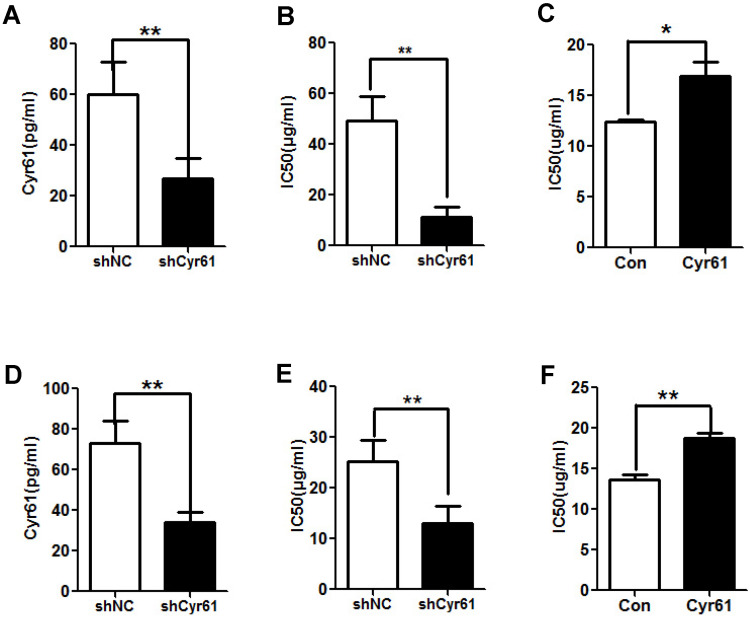
** Cyr61 knockdown reduces the resistance of L-OHP-resistant CRC cells.** (A) and (D) Lentiviral vectors expressing shCyr61 were transduced into HCT-8/L-OHP cells and HCT116/L-OHP cells to knock down Cyr61 expression, and the concentration of Cyr61 in the cell supernatant was detected by ELISA. (B) and (E) shCyr61 and shNC cells from HCT-8/L-OHP cells and HCT116/L-OHP cells were treated with increasing doses of L-OHP for 72 h and subjected to a CCK8 assay; the IC50 of L-OHP in the cells was then calculated using GraphPad Prism 5.0. (C) and (F) HCT-8 and HCT116 cells were preincubated with exogenous Cyr61 (1 µg/ml) for 24 h and were then treated with increasing doses of L-OHP. After incubation for 72 hours and analysis with a CCK8 assay, the IC50 of L-OHP in the cells was calculated using GraphPad Prism 5.0. The data are expressed as the mean ± SEM values (n=3). *P < 0.05, **P < 0.01.

To assess the role of Cyr61 in the resistance of CRC cells to L-OHP, the levels of Cyr61 in HCT-8/L-OHP and HCT116/L-OHP cells were decreased with shCyr61 (Figure 3A and D); these cells were named HCT-8/L-OHP-shCyr61 cells and HCT116/L-OHP-shCyr61 cells, respectively. Then, HCT-8/L-OHP-shCyr61 and HCT116/L-OHP-shCyr61 cells were treated with different concentrations of L-OHP for 72 h. Compared with those in the corresponding shNC cells, the IC50 values of L-OHP in shCyr61 cells were significantly decreased (Figure 3B and E), indicating that Cyr61 knockdown sensitizes L-OHP-resistant CRC cells to L-OHP. Next, HCT-8 and HCT116 cells in suspension culture were exposed to exogenous Cyr61, and its effects on the IC50 values of L-OHP were examined. As shown in Figure 3C and F, the IC50 values of L-OHP in HCT-8 and HCT116 cells were markedly increased under exposure to exogenous Cyr61. These results show that Cyr61 is involved in the resistance of CRC cells to L-OHP.

### Cyr61 knockdown increases the apoptosis of L-OHP-resistant CRC cells induced by L-OHP

**Figure 4:**
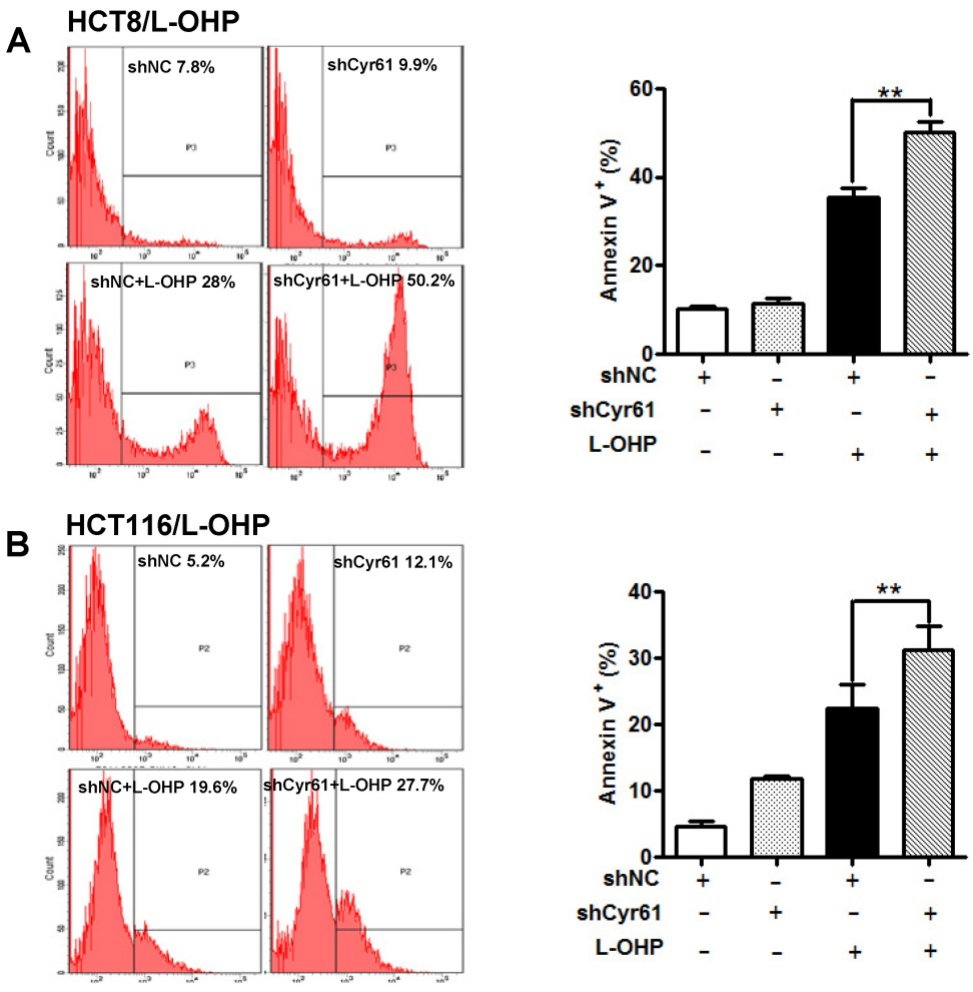
** Cyr61 knockdown enhances L-OHP-induced apoptosis in L-OHP-resistant CRC cells**. (A) HCT-8/L-OHP-shCyr61 and HCT-8/L-OHP-shNC cells were treated with L-OHP (50 µg/ml) for 72 h, and apoptosis was assessed with a BD FACSCanto II cytometer. (B) HCT116/L-OHP-shCyr61 and HCT116/L-OHP-shNC cells were treated with L-OHP (30 µg/ml) for 72 h, and the percentages of apoptotic cells were determined with a BD FACSCanto II cytometer. The data are expressed as the mean ± SEM values (n=3). *P < 0.05, **P < 0.01.

Early studies showed that Cyr61 effectively reduces the chemosensitivity of tumor cells by decreasing the apoptosis induced by chemotherapeutic drugs in various types of cancer, such as breast cancer 5, acute myeloid leukemia 17, chronic myeloid leukemia 10, ovarian cancer 4, acute lymphoblastic leukemia 9, and renal cell carcinoma 18. To investigate the mechanism underlying the Cyr61-induced resistance of CRC cells to L-OHP, we further explored whether Cyr61 knockdown affects apoptosis induced by L-OHP in HCT-8/L-OHP and HCT116/L-OHP cells. The results showed that L-OHP-induced apoptosis was enhanced by knockdown of Cyr61 in HCT-8/L-OHP and HCT116/L-OHP cells (Figure 4A and B), indicating that Cyr61 is involved in the resistance of CRC cells to L-OHP by decreasing L-OHP-induced apoptosis.

### Bcl-xL was involved in the effect of Cyr61 on drug resistance

**Figure 5:**
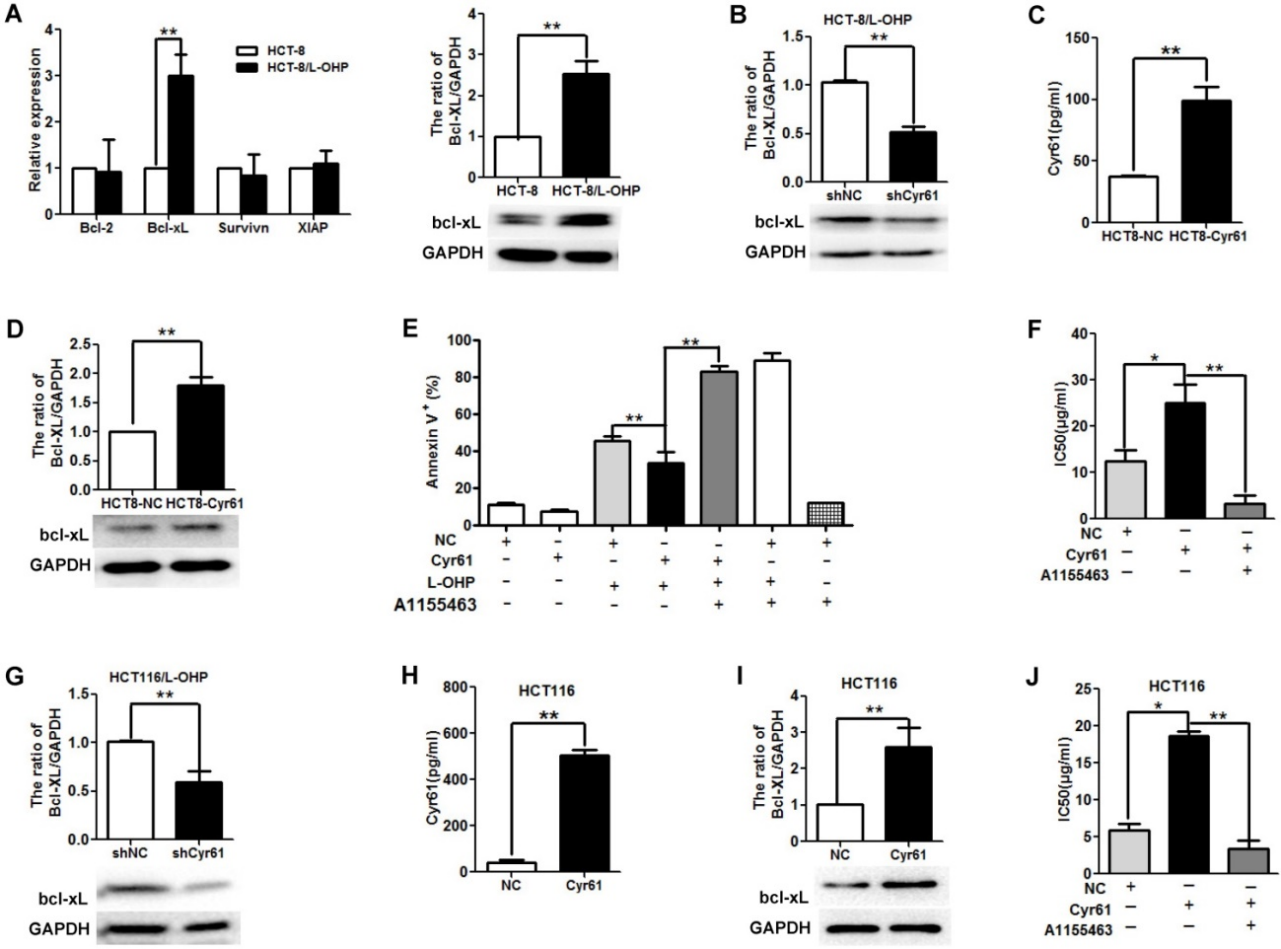
** Bcl-xL is involved in the effect of Cyr61 on drug resistance. (A)** Left panel: Bcl-2, Bcl-xL, XIAP and Survivin mRNA levels in HCT-8/L-OHP and parental HCT-8 cells were determined by real-time PCR; Right panel: Bcl-xL protein levels in HCT-8/L-OHP and parental HCT-8 cells were determined by western blotting. (B) HCT-8/L-OHP-shCyr61 and HCT-8/L-OHP-shNC cells were treated with L-OHP (50 µg/ml) for 72 h, and the protein level of Bcl-xL was detected by western blotting. (C) HCT-8-Cyr61 and HCT-8-NC cells were cultured for 72 h, and the concentration of Cyr61 in the cell supernatant was determined by ELISA. (D) HCT8-Cyr61 and HCT8-NC cells were treated with L-OHP (10 µg/ml) for 72 h, and the protein level of Bcl-xL was determined by western blotting. (E) HCT-8-Cyr61 and HCT-8-NC cells were treated with L-OHP (10 µg/ml), A1155463 (5 µM) (a specific Bcl-xL inhibitor), or L-OHP + A1155463 for 72 h, and apoptosis was analyzed by flow cytometry. (F) Left panel: HCT-8-Cyr61 and HCT-8-NC cells were treated with increasing doses of L-OHP or increasing doses of L-OHP+ A1155463 (5 µM) for 72 h and were then analyzed with a CCK8 assay The IC50 of L-OHP in HCT-8-Cyr61 and HCT-8-NC cells was calculated using GraphPad Prism 5.0. (G) HCT116/L-OHP-shCyr61 and HCT116/L-OHP-shNC cells were treated with L-OHP (30 µg/ml) for 72 h, and the protein level of Bcl-xL was determined by western blotting. (H) HCT116-Cyr61 and HCT116-NC cells were cultured for 72 h, and the concentration of Cyr61 in the cell supernatant was determined by ELISA. (I) HCT116-Cyr61 and HCT116-NC cells were treated with L-OHP (10 µg/ml) for 72 h, and the protein level of Bcl-xL was determined by western blotting. (J) HCT116-Cyr61 and HCT116-NC cells were treated with increasing doses of L-OHP or increasing doses of L-OHP+ A1155463 (5 µM) for 72 h and were then analyzed with a CCK8 assay. Then, the IC50 of L-OHP in HCT116-Cyr61 and HCT116-NC cells was calculated using GraphPad Prism 5.0. The data are expressed as the mean ± SEM values (n=3). *P < 0.05, **P < 0.01.

Bcl-2 family molecules play key roles in cellular apoptosis. Accumulating studies have shown that Cyr61 decreases tumor cell apoptosis induced by chemotherapy by regulating the levels of Bcl-2 family molecules, such as Bcl-2, Bcl-xL, and XIAP 4-5, 10. To explore the molecular mechanism underlying the antiapoptotic effects of Cyr61 in L-OHP-resistant CRC cells, we first evaluated the levels of Bcl-2, Bcl-xL, XIAP, and Survivin; the results showed that the Bcl-xL mRNA and protein levels in HCT-8/L-OHP cells were significantly higher than those in HCT-8 cells, while no significant difference was found in the mRNA levels of Bcl-2, XIAP, and Survivin (Figure 5A). Next, we evaluated the effect of Cyr61 on the expression of Bcl-xL in HCT-8/L-OHP cells. As presented in Figure 5B, knockdown of Cyr61 decreased the level of Bcl-xL in HCT-8/L-OHP cells, suggesting that Cyr61 promotes the expression of Bcl-xL in HCT-8/L-OHP cells.

To further explore whether Bcl-xL is involved in the effect of Cyr61 on drug resistance, HCT-8 cells were transfected with pGLV5-Cyr61 to induce Cyr61 overexpression; these cells were named HCT-8-Cyr61 cells (Figure 5C). The results showed that the expression of Bcl-xL in HCT-8-Cyr61 cells was higher than that in HCT-8-NC cells (Figure 5D). HCT-8-Cyr61 cells were incubated with or without the Bcl-xL inhibitor A1155443 for 72 h, and the results showed that the level of apoptosis induced by L-OHP in HCT-8-Cyr61 cells was lower than that in HCT-8-NC cells; however, blocking Bcl-xL function increased the level of HCT-8-Cyr61 cell apoptosis induced by L-OHP (Figure 5E). In addition, we observed that the IC50 value of L-OHP in HCT-8-Cyr61 cells was significantly increased compared to that in HCT-8-NC cells and that blocking Bcl-xL function with A1155443 decreased the IC50 value of L-OHP in HCT-8-Cyr61 cells (Figure 5F).

To investigate whether the involvement of Bcl-xL in the effect of Cyr61 on drug resistance is common across CRC cells, we carried out the same experiments using another human L-OHP-resistant CRC cell line, HCT116/L-OHP, and the parental HCT116 cells. The results showed that knockdown of Cyr61 decreased the level of Bcl-xL in HCT116/L-OHP cells (Figure 5G), that increasing Cyr61 expression increased the level of Bcl-xL in HCT116 cells (Figure 5H and Figure 5I), and that the IC50 value of L-OHP in HCT-8-Cyr61 cells was significantly increased compared to that in HCT116-NC cells (Figure 5J). In addition, blocking Bcl-xL function with A1155443 decreased the IC50 value of L-OHP in HCT116-Cyr61 cells (Figure 5J).

Collectively, these results provide evidence that L-OHP-resistant CRC cells is caused by Cyr61, possibly through upregulation of Bcl-xL.

## Discussion

An anticancer drug widely used in clinical practice for the treatment of CRC, oxaliplatin (L-OHP) significantly improves the outcome of CRC patients; however, acquired resistance to L-OHP has become an unavoidable problem 2. High expression of Cyr61 in L-OHP-resistant CRC cells was found in this study. Importantly, increased Cyr61 expression promotes CRC cell resistance by inhibiting L-OHP-induced apoptosis and increasing the level of Bcl-xL.

Cyr61 is an important extracellular matrix-associated protein 3. Previous reports have shown that Cyr61 plays a critical role in maintaining normal cellular functions, such as proliferation, migration, and differentiation 3. Recently, an increasing number of studies have shown that Cyr61 is highly expressed and that increased Cyr61 expression not only promotes tumor cell growth and metastasis but also mediates chemotherapeutic resistance in various types of cancer 4-10. In patients with CRC, overexpression of Cyr61 is found in cancer tissues and serum and is related to tumor cell growth and metastasis and shorter survival times 11-15. However, the role of increased Cyr61 expression in the resistance of CRC cells to L-OHP has not been reported.

In the current study, we successfully established two L-OHP-resistant cell lines, HCT-8/L-OHP and HCT116/L-OHP, and found that Cyr61 levels were significantly increased in L-OHP-resistant cells. To explore the role of increased Cyr61 expression in the resistance of CRC cells to L-OHP, we knocked down the expression of Cyr61 in L-OHP-resistant CRC cells and found that Cyr61 knockdown decreased the resistance of CRC cells to L-OHP. These findings indicated that Cyr61 plays a crucial role in CRC cell L-OHP resistance and that Cyr61 might be a possible therapeutic target for overcoming L-OHP resistance in CRC.

As mentioned earlier, Cyr61 promotes tumor cell resistance by decreasing drug-induced apoptosis in various types of cancer 5, 9-10, 17, 19-20. However, the mechanism by which Cyr61 is involved in the resistance of CRC cells to L-OHP is unclear. In this work, we found that Cyr61 knockdown increased L-OHP-induced apoptosis in L-OHP-resistant CRC cells. Previous reports have shown that Bcl-2 family members are key regulators of cellular apoptosis; thus, we explored the role of Cyr61 on the expression of Bcl-2 family proteins, and the results showed that Cyr61 knockdown decreased the level of Bcl-xL in the two L-OHP-resistant CRC cell lines (HCT-8/L-OHP and HCT116/L-OHP) and that Cyr61 overexpression increased the level of Bcl-xL in HCT-8 and HCT116 cells. The signaling pathways by which Cyr61 regulates the expression of Bcl-XL in CRC cells are unclear. Previous studies have shown that Cyr61 endows malignant cells with resistance to chemotherapy by regulating Bcl-2 family molecule expression via the NF-κB 9-10, ERK1/2 19, and PI3K-AKT 21 signaling pathways; thus, we speculated that the Cyr61-induced increase in the Bcl-XL level may be related to the NF-κB, ERK1/2, and PI3K-AKT signaling pathways in CRC cells. However, the signaling pathway by which Cyr61 regulates the expression of Bcl-xL needs further study. Considering that Bcl-xL is an antiapoptotic protein in the Bcl-2 family, these results demonstrated that Cyr61 inhibits L-OHP-induced apoptosis in L-OHP-resistant CRC cells by increasing the Bcl-xL level.

Some limitations of this study should be acknowledged. First, this study only focused on the role of Cyr61 in the resistance of CRC cells to L-OHP. 5-Fluorouracil and leucovorin are also widely used for CRC treatment, and the effect of Cyr61 on the resistance of CRC cells to 5-fluorouracil and leucovorin remains unknown and requires further study. Second, our finding about the effect of Cyr61 on CRC cell resistance was made in two L-OHP-resistant cell lines; thus, further study should be conducted to explore the relationship between Cyr61 levels and drug resistance in CRC patients.

In summary, we are the first to discover that Cyr61 levels are significantly upregulated in L-OHP-resistant CRC cells and that high Cyr61 expression can effectively protect CRC cells from L-OHP-induced apoptosis by increasing Bcl-xL, leading to failure of CRC cells to respond to L-OHP chemotherapy. These results provide evidence that Cyr61 overexpression promotes the resistance of CRC cells to L-OHP, making Cyr61 a promising target for overcoming L-OHP resistance in CRC.

## Abbreviations

Cyr61: cysteine-rich protein 61
L-OHP: oxaliplatin
CRC: colorectal cancer
CCK-8: cell counting Kit-8
HCT-8/L-OHP: HCT-8 cell resistance to L-OHP
HCT116/L-OHP: HCT116 cell resistance to L-OHP
IC50: 50% inhibitory concentration
ELISA: enzyme-linked immunosorbent assay
PVDF: polyvinylidene difluoride
GAPDH: glyceraldehyde 3-phosphate dehydrogenase
SDS-PAGE: sodium dodecyl sulfate polyacrylamide gel electrophoresis
RIPA: radio-immunoprecipitation assay
